# Integrating programme theory into the development of a core outcome set for technology-assisted counselling interventions in dementia: study protocol of the ProCOS study

**DOI:** 10.1136/bmjopen-2023-081526

**Published:** 2024-08-06

**Authors:** Dorothee Bauernschmidt, Janina Wittmann, Anja Bieber, Gabriele Meyer

**Affiliations:** 1 Institute of Health and Nursing Science, Martin Luther University Halle-Wittenberg, University Medicine Halle, Halle, Saxony-Anhalt, Germany

**Keywords:** dementia, health services for the aged, nursing care

## Abstract

**Abstract:**

**Introduction:**

Due to the increasing number of persons with dementia, the need for family and professional support is growing. Counselling services aim to support family dementia caregivers and the use of information and communication technology may improve accessibility to counselling. The effectiveness of technology-based counselling in dementia remains unclear so far. Few randomised controlled trials have been conducted assessing heterogeneous outcomes. Theoretical underpinnings for the development and evaluation of these complex interventions were lacking in most cases. We therefore aim to formulate an initial programme theory of a technology-assisted counselling intervention for family dementia caregivers and to create the data basis for the consensus process of a core outcome set.

**Methods and analysis:**

The methodological approaches for developing a programme theory and a core outcome set will be integrated. In a scoping review, data on the characteristics, theoretical foundations of counselling interventions and outcomes of clinical studies will be collected. The lifeworld perception of relevant stakeholders on the importance of counselling in family caregiving will be explored in a phenomenological substudy using semistructured interviews. The synthesis of data from the literature review and the qualitative substudy will be performed by developing a logic model. Mechanisms of action and assumed causal relationships are explicated in the elements of programme theory (theory of change, outcomes chain and theory of action). An initial programme theory is then formulated. In addition, a ‘long list’ of outcomes and assessment instruments will be compiled.

**Ethics and dissemination:**

The ethics committee of the Medical Faculty of the Martin Luther University Halle-Wittenberg approved the study protocol (no. 2023–093).

Findings will be reported to participants and the funding organisation and disseminated in peer-reviewed journals and at national and international conferences.

**Trial registration number:**

The ProCOS (Development and evaluation of a technology-assissted counselling intervention for family caregivers of persons with dementia - Programme theory and preparation of a core outcome set) project is registered with the Core Outcome Measures in Effectiveness Trials initiative (https://www.comet-initiative.org/Studies/Details/2884).

**STRENGTHS AND LIMITATIONS OF THIS STUDY**

- The phenomenological perspective will enable the exploration of the experiences of persons receiving and delivering counselling in dementia.
- An updated systematic literature search without date restrictions will provide a comprehensive overview of interventions using technology for delivering counselling.
- A novel approach is used to integrate the methodological strands for developing a programme theory and a core outcome set.
- A possible limitation might be that the logic model cannot fully represent the double complexity of interventions.
- It could be judged as a limitation that the consensus process cannot be conducted within the limited timeframe of the ProCOS project.

## Background and rationale

 The number of persons living with dementia worldwide is expected to rise to 152 million by the year 2050.^1^ At some point in the course of the disease, the decline of cognitive functions and behavioural and psychological changes^2^ result in an impaired ability to cope with everyday life and in an increasing need for family support. Due to the physical, mental and financial impact of dementia care,^3^ family caregivers may need professional support, but there are barriers to accessing and using formal care described in the literature.^4^

A common form of support for family caregivers is counselling services. Counselling can be defined as the ‘use of an interactive helping process focusing on the needs, problems, or feelings of the patient and significant others to enhance or support coping, problem solving, and interpersonal relationships’.^5^ Counselling provided by professionals such as nurses, social workers or psychologists is based on multidisciplinary knowledge, and a systematic approach is applied by using instruments such as assessments, guidelines or proposals for individual use.^6^ Counselling can thereby be differentiated from informal problem-solving or supportive conversations, which are intended to facilitate social interaction and mutual learning based on similar experiences.^7^

Information and communication technology (ICT) has been used to improve accessibility to counselling. Telephone helplines have been established decades ago^8^,^10^ and services that use videoconferencing software, email and/or chats to deliver counselling have increasingly appeared in recent years.^11 12^ Due to the SARS-CoV-2 pandemic, the use of ICT in delivering healthcare interventions was discussed more intensively.^13 14^ Technology-based counselling is considered a convenient way to provide services to persons who are homebound or to caregivers who do not have to arrange substitute care when counselling is provided remotely. In addition, persons living in rural areas with limited access to transport options may benefit from the use of ICT.^6 15^ Telephone helplines use a widespread and undemanding technology^16^ and can provide anonymous support.^17^ The lack of visual and non-verbal cues is a limitation of telephone counselling,^18^ which may be overcome with the utilisation of videoconferencing software.^19^ Synchronous videoconferencing facilitates a more direct interaction^19^ but poses challenges in terms of technological requirements and digital literacy.^20^ Asynchronously delivered counselling via email enables consumers to access services at any time but may prevent individuals from expressing themselves fully.^21 22^

The effectiveness of technology-based counselling in dementia has not yet been proven.^23^ We conducted a systematic review and included five randomised controlled trials. Meta-analyses revealed no significant effects of technology-based counselling interventions on depressive symptoms, burden and self-efficacy/mastery perceived by family caregivers of persons with dementia.^23^ Individual studies suggest some beneficial effects on outcomes, such as caregiver reaction to dementia-related behaviour and resource use.^23^ A wide range of outcomes (n=14) was examined in studies using different assessment instruments (n=21).^23^ In most cases, we found no theoretical basis guiding the development and evaluation of these complex interventions.^23^

The heterogeneity in outcomes examined in clinical studies and lack of theoretical foundation can be addressed in different ways:

The use of a core outcome set (COS) can reduce heterogeneity between trials and enhance comparability and thus, synthesis of evidence.^24^ A COS is ‘an agreed standardised collection of outcomes which should be measured and reported, as a minimum, in all trials for a specific clinical area’.^24^

COS for healthcare interventions in dementia, listed in the database of the Core Outcome Measures in Effectiveness Trials (COMET) initiative,^25^ predominately focus on outcomes of persons with dementia, as does the COS for the evaluation of non-pharmacological community-based health and social care interventions for people with dementia living at home.^26^ In addition, a set of measures has been recommended to evaluate psychosocial interventions for persons with dementia and their family caregivers.^27^

Non-pharmacological health and social care interventions include psychosocial and psychological interventions, educational and social programmes, case management and care coordination as well as assistive technology.^26^ Psychosocial interventions may address persons with dementia, family caregivers or both and are derived from diverse theories targeting a broad range of outcomes such as well-being, mood or behaviour.^28^ A COS that specifically focuses on technology-assisted counselling interventions for family dementia caregivers may contribute to assess the effectiveness of these interventions and to fill this gap in knowledge. In addition, outcomes associated with the use of ICT in delivering counselling may be identified in the developmental process. By drawing on existing COS, we assume that there will be overlapping domains but also areas, which may be complementary.

Theory-led approaches are essential to successfully develop, implement and evaluate complex interventions. This has been highlighted by the Framework for Developing and Evaluating Complex Interventions, which identifies programme theory as a core element of complex interventions.^29^ A programme theory is an ‘explicit theory of how an intervention is understood to contribute to its intended or observed outcomes’.^30^ The explication of assumed causal relationships and mechanisms of action allows for the quality of the theory to be critically reviewed, fosters a shared understanding among stakeholders and guides the implementation and evaluation of the intervention.^29 30^

In the ProCOS study, we build on previous work,^31^ which focused on the effectiveness^23^ and the implementation success^6^ of technology-based counselling interventions. To integrate findings of different modalities in delivering counselling, we differentiated the following types of interventions: counselling via telephone or email and counselling via videoconferencing; web-based psychosocial intervention: information, communication and counselling; videoconference or telephone-based counselling combined with tele-monitoring or psychoeducation and technology-based counselling as part of a comprehensive programme with non-technology-based components.^6^ By updating the former literature search,^31^ we expand the data basis to address the shortcomings of previous research described in the preceding sections. Therefore, we aim at bringing together the developmental processes of a COS and a programme theory for technology-assisted counselling interventions for family caregivers of persons with dementia.

There are two sets of questions guiding the research interest and informing the selection of methods within the ProCOS study:

1. What interventions that use ICT to provide counselling for family dementia caregivers are described in literature? What are the characteristics (frequency and duration, type of technology used, components of the interventions, stakeholders involved, adaptations to the context) of these interventions? What theoretical underpinnings for intervention development and implementation are explicated in the form of theoretical references, programme theories and/or logic models? What outcomes have been examined in clinical trials? What assessment instruments have been used?
2. How do family caregivers for persons with dementia and counsellors experience counselling services? What mutual expectations have persons seeking or providing counselling? Which outcomes should or could be achieved through counselling, and how can these outcomes be achieved? Which factors have an impact on the effectiveness of counselling? What are appropriate outcomes for assessing the effectiveness of counselling interventions?

## Methods and analysis

### Study design

**Figure 1:**
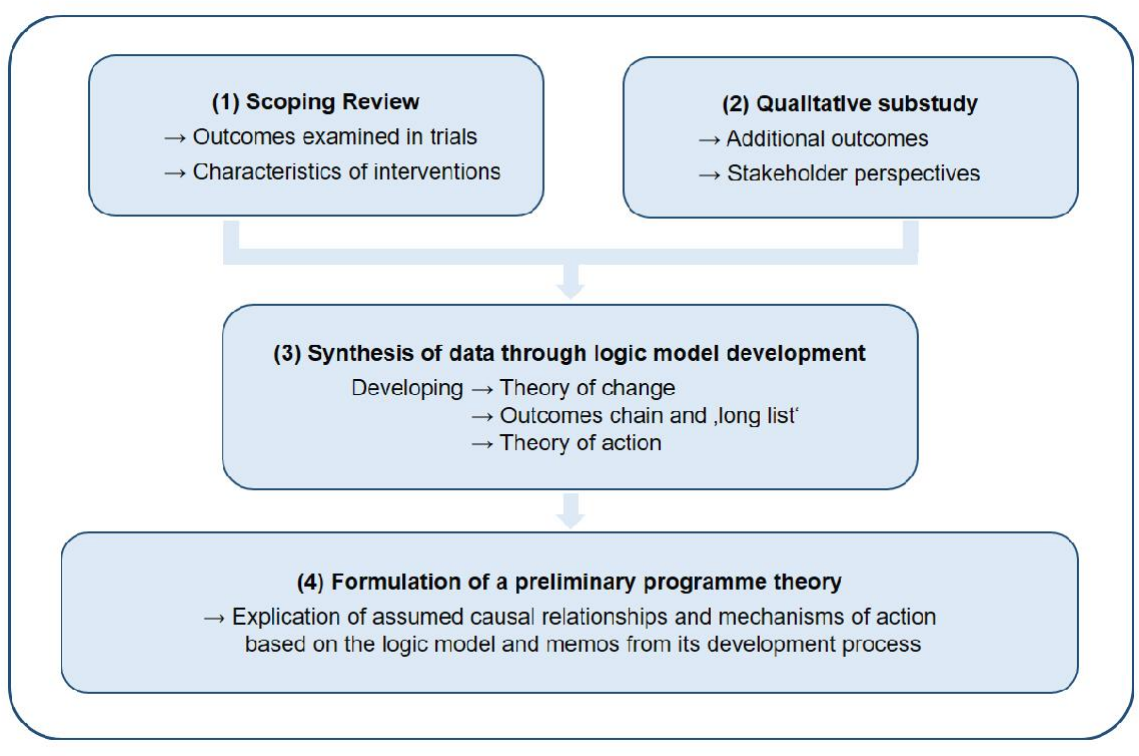
Working steps within the ProCOS study.

To answer the aforementioned questions, a literature review and a qualitative substudy will be conducted. Results will be synthesised by developing a logic model, which comprises the elements of a ‘purposeful program theory’.^30^ Mechanisms of how changes can be achieved are described in the theory of change. The outcomes chain illustrates (possible) outcomes of the intervention, thereby constituting the ‘long list’ for the COS consensus process. The theory of action delineates what will be done within the programme or intervention to activate the theory of change and to achieve the outcomes.^30^ Based on the logic model, the preliminary programme theory of a technology-assisted counselling intervention in dementia will be formulated. A graphical presentation of the four working steps is displayed in figure 1.

The Preferred Reporting Items for Systematic reviews and Meta-Analyses extension for Scoping Reviews,^32^ the Standards for Reporting Qualitative Research^33^ and the COS-STAndardised Protocol Items: the COS-STAP Statement^34^ were used to structure this protocol. Populated checklists are provided in the online supplemental file 1.

### Scoping review

To map the evidence on characteristics and theoretical foundations of counselling interventions as well as the outcomes examined in clinical studies, a scoping review will be conducted following the Joanna Briggs Institute methodological guidance.^35^ This approach was chosen based on the definition that characterises scoping reviews as a type of evidence synthesis aiming ‘to systematically identify and map the breadth of evidence available on a particular topic, field, concept, or issue, often irrespective of source’.^36^

#### Eligibility criteria

Studies on counselling interventions for caregivers of persons with dementia will be included, irrespective of their design. Due to language capacities of the research team, we will include publications written in English or German. Table 1 displays the inclusion and exclusion criteria according to the PCC scheme (population, concept and context).^35^

**Table 1:** Inclusion and exclusion criteria according to the PCC scheme^35^

	Inclusion	Exclusion
Population	Family caregivers of persons with dementia	Professional caregivers
Concept	Tailored and individualised counselling on various issues in caring for persons with dementia (such as dealing with behavioural changes, coping strategies, reconciling caregiving responsibilities with family and/or professional engagement, available support and transition to nursing home)	Therapeutical approaches (such as psychotherapy, cognitive-behavioural therapy);standardised counselling interventions;interventions exclusively delivering information/education andcounselling on diagnostics or genetic issues
	Counselling is provided via ICT (combined with personal contacts)	Counselling is provided via personal contacts exclusively
Context	Home care arrangements andinstitutional care	No limitations

ICTinformation and communication technologyPCCpopulation, concept and context

#### Information sources and search strategy

The databases CINAHL, MEDLINE, Cochrane Library and PsycINFO will be searched in combination with forward and backward citation tracking.^37^ In addition, free web searching via Google and Google Scholar will be conducted in order to identify grey literature, such as reports. For non-published material such as manuals, handbooks and training materials, authors will be personally approached.

We will update the literature search of a previous systematic review.^31^ This search was conducted without date restrictions, and the database-specific search strategies as well as the search terms of the free web search are provided elsewhere.^23^

#### Selection of sources of evidence

Screening of titles, abstracts and full texts will be performed independently by two researchers using the Rayyan web app.^38^ Discrepancies in decisions will be resolved by discussion.

#### Data charting process and data items

An extraction sheet will be developed based on the previous work. Data on study characteristics (year of publication, country of study conduct, design and methods and number of participants), outcomes examined and characteristics of interventions will be extracted. Criteria from the Template for Intervention Description and Replication checklist^39^ and from the revised Criteria for Reporting the Development and Evaluation of Complex Interventions guideline^40^ will be applied to extract information on objectives, components, theoretical underpinnings of counselling interventions as well as technology and materials used for delivering counselling and frequency/duration of sessions. Data extraction will be performed by one reviewer and cross-checked by another researcher.

### Qualitative substudy

By this substudy, we aim to explore the lifeworld perception of relevant stakeholders on the importance of counselling in family caregiving from a phenomenological perspective.

#### Qualitative approach and research paradigm

Phenomenology is an approach ‘where the subject is understood as an embodied and socially and culturally embedded being-in-the-world’.^41^ A central concept of phenomenology is that of the lifeworld, which is understood as the world we take for granted in everyday life and that we do not question.^41^ Based on Schütz’s phenomenological sociology, we will focus on the essential structures of participants’ lifeworld.^42^

#### Context, researcher characteristics and reflexivity

The study will be conducted at an institute with a focus on dementia research. Assumptions and presuppositions resulting from the authors’ long-term engagement in this research field will be reflected and disclosed. The primary investigator has had experience in phenomenological research. This approach was chosen for the substudy because it allows the exploration of the lived experience of family dementia caregivers who have received counselling, and of persons who are providing counselling.

#### Units of study and sampling strategy

Interviews will be conducted with family dementia caregivers and persons who provide counselling in the field of family dementia care. To gain a deeper understanding of different approaches in the provision of counselling, we will include persons who have received or delivered counselling via technology, in person or both. Participants will be recruited by purposive sampling.^43^ A variance regarding the caregivers’ characteristics (age, gender, socioeconomic status, duration of caregiving and relationship to the care-receiving person) and those of the counsellors (professional qualifications and socialisation, duration of occupational experience, characteristics of employing organisations such as welfare organisations, municipal and private providers and healthcare insurances) is intended. Caregivers living with or near the person with dementia as well as long-distance caregivers will be included. Underaged persons (under 18 years old) and persons unable to consent for language reasons will not be included.

We will draw on existing contacts and networks to get access to the research field. We intend to recruit about 15 caregivers of persons with dementia and about 10 persons delivering counselling. The final sample size will be determined by saturation of information during the data collection process.^44^

#### Data collection methods and data processing

Semistructured interviews will be conducted.^45^ Open-ended questions will be asked in order to prompt narratives and to give the interviewees room to share their experiences.^45^ Questions will address experiences in receiving and providing counselling as well as stakeholders’ mutual expectations. Sociodemographic characteristics and information on the care arrangement and professional situation will be collected. Time and place of the interviews will be arranged at participants’ convenience. All interviews will be recorded and transcribed verbatim.

#### Data analysis

**Figure 2:**
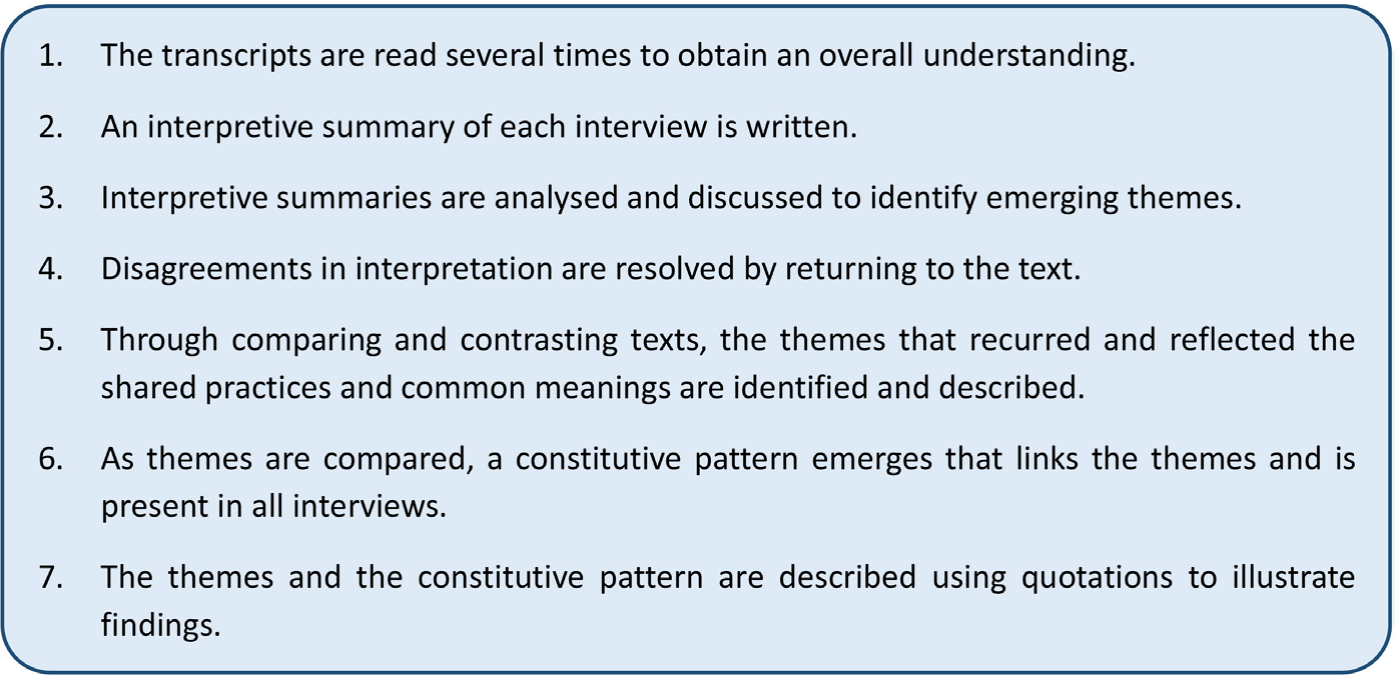
Stages of the interpretive phenomenological analysis, modified after Diekelmann.^46^

An interpretive phenomenological analysis will be performed applying a modified version of the seven-stage process described by Diekelmann.^46^ The analysis aims to identify themes representing shared practices and common meanings in participants’ lifeworld. Stages of analysis are displayed in figure 2.

### Synthesis of data through logic model development

The synthesis of data from the literature review and the qualitative substudy will be performed through the development of a logic model. Logic models are used for data synthesis in systematic reviews and are suitable for mapping the complexity of interventions and promoting conceptual thinking.^47^,^49^ Data extracted from included publications of the scoping review will be treated as qualitative data^50^ and synthesised with findings from the interpretive phenomenological analysis. Methods such as charting and categorising^50^ as well as thematic synthesis^51^ will be applied to assign data to the elements of a programme theory.

The elements of programme theory are represented in the logic model: the theory of change explains the central mechanism of how the intended changes can be achieved and the theory of action describes how the intervention is designed to initiate the theory of change. These elements are linked by the outcomes chain, which maps the immediate and intermediate outcomes and ultimate impacts as well as the assumed or hypothesised relationships between outcomes.^30^

The developmental process of the logic model draws on a variety of published examples and templates^30 52 53^ and will be accompanied by continuous documentation (memos) which will be used for formulating the preliminary programme theory.

The starting point in developing the theory of change is a situation analysis of the nature and scope, causes, influencing factors and consequences of the problem to be addressed by the intervention. Questions described by Funnell and Rogers^30^ will be applied to guide the situation analysis.

In the second step, the desired and intended outcomes will be identified. The identification of relevant outcomes is based both on the procedure recommended by Funnell and Rogers^30^ and in the COS methodology^24^ on a list of outcomes ('long list'), which is compiled on the basis of literature and supplemented by additional outcomes named by stakeholders.^54^ In the ProCOS study outcomes extracted within the scoping review will be supplemented by outcomes provided by participants of the qualitative substudy. Challenging here is the translation of the interviewees’ statements into clinical outcomes^24^ in order to include them as accessible statements^55^ in the subsequent consensus-building process. To adequately address the stakeholder perspectives, the members of the study advisory board will be consulted.

The outcomes of the ‘long list’ will be then assigned to domains^24^ and structured by distinguishing short-term and long-term outcomes,^30^ thus forming the chain of outcomes of the intervention.

From these two steps, the theory of action will be designed. For this purpose, the characteristics of the intended outcomes as well as unintended consequences will be described, influencing factors identified and activities defined that are implemented to achieve the outcomes.^30^ The key is to define criteria of success to make the effects of an intervention visible or measurable and to designate measures of how these effects are to be achieved.^30^ The outcomes of the outcomes chain are specified with attributes on quality, quantity and timeliness, among others, and it is determined what, when, where, how, why and for whom is to be achieved.^30^ Another important factor in determining effectiveness is the definition of comparisons, for which norms and standards or the extent of change over time can be used.^30^

### Formulation of a preliminary programme theory

An initial programme theory will be formulated based on the logic model and the memos produced during its development process. Theories and concepts identified in the extracted data of the scoping review are included. Causality assumptions and mechanisms of action as well as interactions among intervention components^29^ will be explicated. Aspects significant to the implementation of the intervention will also be included in the programme theory.

### Patient and public involvement

A study advisory board will be established. Persons with experience in caring for a family member with dementia and in delivering counselling, as well as an experienced dementia researcher will be recruited based on the networks from previous projects and from a caregiver support group moderated by a researcher from the Institute of Health and Nursing Science in Halle (Saale). These persons will act as advisory board members throughout the project. As a co-production team representing the perspectives of different stakeholder groups, the advisory board members will be involved in appraising the adequacy and feasibility of approaches to data collection and in reviewing the results. Meetings will be scheduled prior to data collection, during and after completion of the analysis in order to obtain feedback on the planned procedures and (interim) results. In advance, emails announcing topics and the expected duration of the planned meetings will be sent. The members of the study advisory board will also be engaged in the dissemination of study findings.

### Techniques to enhance trustworthiness

By consulting the members of the study advisory board, strategy expert consultation and peer debriefing will be applied to enhance the trustworthiness of the findings. Experts and peer researchers will be involved throughout all stages of the research process, that is, to review the research questions and the interview guide as well as the findings of the analysis and the results of the data synthesis.

### Ethics and dissemination

The ProCOS study has been approved by the ethics committee of the Medical Faculty of the Martin Luther University Halle-Wittenberg (no. 2023–093).

Persons interested in participating will be informed of the procedures at the first contact and given time to decide whether or not to participate. Written informed consent will be obtained at the time of the interview and participants will be informed that the consent to participate can be withdrawn at any time.

Security of data will be maintained. Data will be stored in a secure setting, and audio recordings will be pseudonymised during the transcription process.

The findings of the ProCOS study will be reported to all participants and the funding organisation. Results will be disseminated by peer-reviewed international journals and by presentations at national and international conferences. By actively engaging the advisory board members, study findings will also be presented to stakeholder groups to reach a wider audience.

## Discussion

The ProCOS study focuses on technology-assisted counselling for family caregivers of persons with dementia—a support service likely to become increasingly important due to the rising number of persons with dementia and the growing support needs, the regional differences in the availability of support services and the advancing digitalisation.

Methodological approaches in the ProCOS study follow accepted guidelines.^24 30^ In trying to address the shortcomings of previous research, the methodological strands for developing a programme theory and a COS will be integrated. This innovation is a strength of this study, ensuring a theory-led approach to the development, implementation and evaluation of a technology-assisted counselling intervention for family caregivers of persons with dementia.^56^

Logic models have been used to synthesise data in systematic reviews,^47^,^5057^ and as a visualisation of programme theory^29 30^, they represent a core element in developing and evaluating complex interventions.^29^ It has been debated whether logic models can represent the double complexity resulting from interacting components of complex interventions and adaptations of interventions to the context.^58^ Methodological approaches to develop more dynamic logic models have been proposed,^52^ and we will reflect on possible limitations in using logic models to capture double complexity.

Expected results include a systematic overview of the components of counselling interventions and their intended effects as well as the theoretical foundations. Apart from this, a compilation of outcomes (‘long list’) will be created, summarising outcomes already examined and outcomes suggested by the stakeholders interviewed. Furthermore, commonalities and differences in the stakeholder subjective perspectives and relevance structures will become visible.

The results of the ProCOS study will form the basis for a consensus process, which is to be conducted in a follow-up project. Using the Delphi methodology, relevant stakeholders will be involved in determining the important clinical outcomes and critically reviewing the preliminary programme theory. The instrument used in the consensus process is the logic model. Logic models are considered suitable for facilitating communication among stakeholders.^47^ The elements of the logic model will be gradually integrated into the consensus process of the COS so that the selection of appropriate outcomes is informed by stakeholder perspectives and theory—a requirement which has been formulated in the Framework for Developing and Evaluating Complex Interventions.^29^

The finalised programme theory will guide the development and implementation of a technology-assisted counselling intervention for family caregivers of persons with dementia. The consented COS will be used to assess the effectiveness of the intervention and may inform further research in the area of technology-assisted counselling in dementia.

## supplementary material

10.1136/bmjopen-2023-081526 online supplemental file 1
