# Supplementary material for: Integrating programme theory into the development of a core outcome set for technology-assisted counselling interventions in dementia: study protocol of the ProCOS study
Source: BMJ Open. 2024 Aug 6;14(8):e081526. doi: 10.1136/bmjopen-2023-081526 (PMC11308905; doi:10.1136/bmjopen-2023-081526)
Supplement: Supplementary file 2 [file bmjopen-2023-081526.reviewer_comments.pdf]

PEER REVIEW HISTORY

BMJ Open publishes all reviews undertaken for accepted manuscripts. Reviewers are asked to complete a checklist review form (**http://bmjopen.bmj.com/site/about/resources/checklist.pdf**) and are provided with free text boxes to elaborate on their assessment. These free text comments are reproduced below.

ARTICLE DETAILS

**TITLE (PROVISIONAL)**	Integrating programme theory into the development of a Core Outcome Set for technology-assisted counselling interventions in dementia – study protocol of the ProCOS study
**AUTHORS**	Bauernschmidt, Dorothee; Wittmann, Janina; Bieber, Anja; MEYER, Gabriele

VERSION 1 – REVIEW

**REVIEWER**	Brooks, Deborah<br>University of Queensland Centre for Clinical Research
**REVIEW RETURNED**	22-Nov-2023

**GENERAL COMMENTS**	This is a well written and interesting protocol of a study to integrate programme theory into the development of a Core Outcome Set for technology-assisted counselling interventions in dementia. There are some areas for which further clarification would benefit the manuscript.<br>Background<br>Some discussion of how technology-assisted counselling differs from in-person counselling is warranted. In particular, factors influencing the effectiveness or lack of effectiveness of technology-assisted counselling compared to in-person counselling; barriers and facilitators to the receipt and delivery of technology-assisted counselling approaches specific to carers of people living with dementia. How this may impact a specific core outcome set (COS) for technology-assisted counselling interventions for carers of people with dementia (noting that a COS has been developed for non-technology-assisted interventions).<br>How did the authors' prior systematic review (and how will the proposed study), examine / integrate the different technology-assisted delivery modalities i.e. telephone or videoconferencing or text or online chat?<br>How does the proposed study relate to, build upon or differ to the authors' other published protocol: Hirt J, Langer G, Wilde F, Bauernschmidt D, Meyer G, Bieber A. Technology-based counselling in dementia (TeCoDem): study protocol of a mixed-methods systematic review with qualitative comparative analysis and meta-analysis. BMJ Open. 2021 Dec 8;11(12):e054157. doi: 10.1136/bmjopen-2021-054157. PMID: 34880025; PMCID: PMC8655568.<br>Methods<br>Please clarify why the study lends itself to a scoping review approach (item 8 of the PRISMA-ScR checklist).<br>Is the initial search strategy available for review?<br>What is the date range for the review searches, or is it unrestricted?<br>Please spell out JBI (first use) on p5 line 56<br>For the qualitative study, please clarify if the focus (aims, sampling

	methods, interview questions) is on stakeholder experiences of receiving or delivering technology-assisted counselling rather than in-person counselling. The former would fit with the aims of the study and the synthesis for the proposed logic model; however, this is not clearly described. Is there an interview guide available for review?<br>p9 line 11 refers to a fourth work package, however work packages have not been mentioned prior to this. Please clarify work packages 1-3 if retaining this.<br>.

**REVIEWER**	Wong, Karen Lok Yi<br>The University of British Columbia
**REVIEW RETURNED**	02-Dec-2023

**GENERAL COMMENTS**	It was my pleasure to review this protocol. Thank you. The following comments are for your consideration:<br><br>1. Counselling is a key term of this protocol. While I appreciate you defined it, I recommend you consider an elaboration of the definition. For example, what are the differences between professional counseling services and informal problem-solving conversations?<br><br>2. Lifeword is another key term of this protocol. I recommend you consider elaborating on it more.<br><br>3. Why would you only include studies in English and German? Is it because of the language capacity of the team?<br><br>4. I appreciate you mentioned how you are going to search for academic and non-published literature. May I know if you have plans on searching for grey literature that is published (e.g., reports)?<br><br>5. I am glad to know that you are going to involve people with lived experiences as co-researchers. It would be great if you could mention how you recruited them. For example, in some cities in Canada, health authorities have departments promoting community research and groups of people with lived experiences interested in research.<br><br>6. It would be helpful if you could provide more details on how you will involve these co-researchers with lived experiences. For example, will you have meetings with them? What strategies are you going to use to facilitate the discussions?<br><br>Thank you again for inviting me to review your protocol.

**REVIEWER**	Harding, Andrew<br>Lancaster University, Faculty of Health & Medicine
**REVIEW RETURNED**	19-Jan-2024

**GENERAL COMMENTS**	Thank you for asking me to review this protocol manuscript which presents a study that seeks to integrate a programme theory into the development of a core outcome set focusing on technology-assisted counselling interventions in dementia. This is an interesting integration of two research strands, and to my knowledge is novel and interesting. More specifically, this manuscript highlights the study design behind the generation of the long-list and initial programme theory.

	I read this paper with interest and I'm sure it will be an interesting and worthwhile study, particularly as the integration of programme theory and core outcome sets is novel. I only have some minor comments which I think will improve this protocol further.<br><br>1. One page 3 line 41 in reference 16 you cite a COS for psychosocial interventions. Please consider also acknowledging that a COS has been developed on non-pharmacological interventions for people living with dementia – see Siobhan T Reilly, Andrew J E Harding, Hazel Morbey, Faraz Ahmed, Paula R Williamson, Caroline Swarbrick, Iracema Leroi, Linda Davies, David Reeves, Fiona Holland, Mark Hann, John Keady, What is important to people with dementia living at home? A set of core outcome items for use in the evaluation of non-pharmacological community-based health and social care interventions, Age and Ageing, Volume 49, Issue 4, July 2020, Pages 664–671, https://doi.org/10.1093/ageing/afaa015 . This is the output from the research you cited on developing accessible statements (reference 39).<br><br>2. You may wish to consider whether your COS could be considered a bolt on to the one above, as I am aware researchers in more specific fields are currently doing this (and this is being encouraged by COMET).<br><br>3. The authors may wish to elaborate on their definition of 'minors' on page 6, line 35.<br><br>4. The authors may wish to consider giving some indication as to how many participants they intend to recruit for the qualitative sub-study.<br><br>5. The discussion highlights what the next project will do, namely attain consensus on the long-list and programme theory. I would value a more thorough discussion, including on strengths/limitations, and how the outputs from this fuller programme of work (i.e. when the programme theory and COS are fully developed) will inform further research.<br><br>I wish the authors all the best with their submission and undertaking this interesting study.

VERSION 1 – AUTHOR RESPONSE

Reviewer 1:
Dr. Deborah Brooks, University of Queensland Centre for Clinical Research

Comment 1:
Background
Some discussion of how technology-assisted counselling differs from in-person counselling is warranted. In particular, factors influencing the effectiveness or lack of effectiveness of technology-assisted counselling compared to in-person counselling; barriers and facilitators to the receipt and delivery of technology-assisted counselling approaches specific to carers of people living with dementia.

Response:
Thank you for this suggestion. We have included a section on how technology-based counselling differs from in-person counselling.

Changes in the manuscript (background):
Technology-based counselling is considered a convenient way to provide services to persons who are homebound or to caregivers who do not have to arrange substitute care when counselling is provided remotely. In addition, persons living in rural areas with limited access to transport options may benefit from the use of ICT [6, 15]. Telephone helplines use a widespread and undemanding technology [16] and can provide anonymous support [17]. The lack of visual and nonverbal cues is a limitation of telephone counselling [18], which may be overcome with the utilisation of videoconferencing software [19]. Synchronous videoconferencing facilitates a more direct interaction [19] but poses challenges in terms of technological requirements and digital literacy [20]. Asynchronously delivered counselling via email enables consumers to access services at any time but may prevent individuals from expressing themselves fully [21, 22].

Comment 2:
How this may impact a specific core outcome set (COS) for technology-assisted counselling interventions for carers of people with dementia (noting that a COS has been developed for non-technology-assisted interventions).

Response:
We appreciate this comment. A section on how differences may impact a COS specifically focusing on technology-assisted counselling is now part of the manuscript.

Changes in the manuscript (background):
Non-pharmacological health and social care interventions include psychosocial and psychological interventions, educational and social programmes, case management and care coordination as well as assistive technology [26]. Psychosocial interventions may address persons with dementia, family caregivers, or both and are derived from diverse theories targeting a broad range of outcomes such as well-being, mood, or behaviour [28]. A COS that specifically focuses on technology-assisted counselling interventions for family dementia caregivers may contribute to assess the effectiveness of these interventions and to fill this gap in knowledge. In addition, outcomes associated with the use of ICT in delivering counselling may be identified in the developmental process. By drawing on existing COS, we assume that there will be overlapping domains but also areas, which may be complementary.

Comment 3+4:
How did the authors' prior systematic review (and how will the proposed study), examine / integrate the different technology-assisted delivery modalities i.e. telephone or videoconferencing or text or online chat?
How does the proposed study relate to, build upon or differ to the authors' other published protocol: Hirt J, Langer G, Wilde F, Bauernschmidt D, Meyer G, Bieber A. Technology-based counselling in dementia (TeCoDem): study protocol of a mixed-methods systematic review with qualitative comparative analysis and meta-analysis. BMJ Open. 2021 Dec 8;11(12):e054157. doi: 10.1136/bmjopen-2021-054157. PMID: 34880025; PMCID: PMC8655568.

Response:
We have elaborated the section about previous work and on how we integrated findings across different modalities in delivering counselling. A passage has been added.

Changes in the manuscript (background):
In the ProCOS study, we build upon previous work [31], which focused on the effectiveness [23] and the implementation success [6] of technology-based counselling interventions. To integrate findings of different modalities in delivering counselling, we differentiated the following types of interventions: counselling via telephone or email and counselling via videoconferencing; web-based psychosocial intervention: information, communication, and counselling; videoconference or telephone-based counselling combined with tele-monitoring or psychoeducation; and technology-based counselling as part of a comprehensive programme with nontechnology-based components [6]. By updating the former literature search [31], we expand the data basis to address shortcomings of previous research described in the preceding sections. Therefore, we aim at bringing together the developmental processes of a COS and a programme theory for technology-assisted counselling interventions for family caregivers of persons with dementia.

Comment 5:
Methods
Please clarify why the study lends itself to a scoping review approach (item 8 of the PRISMA-ScR checklist).

Response:
Thank you for this suggestion. We have outlined the rationale in more detail in the manuscript.

Changes in the manuscript (methods and analysis):
This approach was chosen based on the definition that characterises scoping reviews as a type of evidence synthesis aiming “to systematically identify and map the breadth of evidence available on a particular topic, field, concept, or issue, often irrespective of source” [36].

Comment 6+7:
Is the initial search strategy available for review?
What is the date range for the review searches, or is it unrestricted?

Response:
Thank you for thesetwo comments, which we have addressed in one section in the manuscript.

Changes in the manuscript (methods and analysis):
We will update the literature search of a previous systematic review [31]. This search was conducted without date restrictions and the database-specific search strategies as well as the search terms of the free web search are provided elsewhere [23].

Comment 8:
Please spell out JBI (first use) on p5 line 56

Response:
We have spelled out the abbreviation.

Changes in the manuscript (methods and analysis):
Joanna Briggs Institute (JBI)

Comment 9:
For the qualitative study, please clarify if the focus (aims, sampling methods, interview questions) is on stakeholder experiences of receiving or delivering technology-assisted counselling rather than in-person counselling. The former would fit with the aims of the study and the synthesis for the proposed logic model; however, this is not clearly described.

Response:
Thank you for pointing this out. In order to get a comprehensive understanding of stakeholders' perspectives on different modalities, we will include persons who have received or delivered counselling via technology, in person, or both.

Changes in the manuscript (methods and analysis):
To gain a deeper understanding on different approaches in the provision of counselling, we will include persons who have received or delivered counselling via technology, in person, or both.

Comment 10:
Is there an interview guide available for review?

Response:
We have developed a first draft of an interview guide, which will be critically reviewed by the members of the study advisory board. The revised interview guide will be made available with the publication of the results.

Changes in the manuscript:
None

Comment 11:
p9 line 11 refers to a fourth work package, however work packages have not been mentioned prior to this. Please clarify work packages 1-3 if retaining this.

Response:
Thank you for pointing this out. We now use the term 'working steps', which is also used in Figure 1. Subheadings of workings steps are numbered accordingly. The phrase 'Within the fourth work package' has been deleted.

Changes in the manuscript (methods and analysis):
A graphical presentation of the four working steps is displayed in Figure 1.

Reviewer: 2
Ms. Karen Lok Yi Wong, The University of British Columbia

Comment 1:
1. Counselling is a key term of this protocol. While I appreciate you defined it, I recommend you consider an elaboration of the definition. For example, what are the differences between professional counseling services and informal problem-solving conversations?

Response:
Thank you for this suggestion. We have elaborated the definition of counselling and the differences between professional counselling and informal problem-solving conversations.

Changes in the manuscript (background):
Counselling provided by professionals such as nurses, social workers or psychologists is based on multi-disciplinary knowledge, and a systematic approach is applied by using instruments such as assessments, guidelines, or proposals for individual use [6]. Counselling can thereby be differentiated from informal problem-solving or supportive conversations, which are intended to facilitate social interaction and mutual learning based on similar experiences [7].

Comment 2:
2. Lifeword is another key term of this protocol. I recommend you consider elaborating on it more.

Response:
Thank you for this comment. We have now included a passage on this term.

Changes in the manuscript (methods and analysis):
A central concept of phenomenology is that of the lifeworld, which is understood as the world we take for granted in everyday life and that we do not question [41].

Comment 3:
3. Why would you only include studies in English and German? Is it because of the language capacity of the team?

Response:
We have added the reason for this decision.

Changes in the manuscript (methods and analysis):
Due to language capacities of the research team, we will include publications written in English or German.

Comment 4:
4. I appreciate you mentioned how you are going to search for academic and non-published literature. May I know if you have plans on searching for grey literature that is published (e.g., reports)?

Response:
Thank you for this comment. We have added a passage on free web searching.

Changes in the manuscript (methods and analysis):
The databases CINAHL, MEDLINE, Cochrane Library and PsycINFO will be searched in combination with forward and backward citation tracking [37]. In addition, free web searching via Google and Google Scholar will be conductedin order to identify grey literature, such as reports.

Comment 5:
5. I am glad to know that you are going to involve people with lived experiences as co-researchers. It would be great if you could mention how you recruited them. For example, in some cities in Canada, health authorities have departments promoting community research and groups of people with lived experiences interested in research.

Response:
We appreciate this feedback. A passage on the recruitment of persons with lived experience as co-researchers has been added.

Changes in the manuscript (methods and analysis):
A study advisory board will be established. Persons with experiencein caring for a family member with dementia and in delivering counselling as well as an experienced dementia researcher will be recruited, based on networks from previous projects and from a caregiver support group moderated by a researcher from the Instituteof Health and Nursing Science in Halle (Saale). These persons will act as advisory board members throughout the project.

Comment 6:
6. It would be helpful if you could provide more details on how you will involve these co-researchers with lived experiences. For example, will you have meetings with them? What strategies are you going to use to facilitate the discussions?

Response:
More details on the involvement of co-researches are included now.

Changes in the manuscript (methods and analysis):
Meetings will be scheduled prior to data collection, during and after completion of the analysis in order to obtain feedback on the planned procedures and (interim) results. In advance, emails announcing topics and expected duration of the planned meetings will be sent.The members of the study advisory board will also be engaged in the dissemination of study findings.

Reviewer: 3
Mr. Andrew Harding, Lancaster University

Comment 1:

1. One page 3 line 41 in reference 16 you cite a COS for psychosocial interventions. Please consider also acknowledging that a COS has been developed on non-pharmacological interventions for people living with dementia – see Siobhan T Reilly, Andrew J E Harding, Hazel Morbey, Faraz Ahmed, Paula R Williamson, Caroline Swarbrick, Iracema Leroi, Linda Davies, David Reeves, Fiona Holland, Mark Hann, John Keady, What is important to people with dementia living at home? A set of core outcome items for use in the evaluation of non-pharmacological community-based health and social care interventions, Age and Ageing, Volume 49, Issue 4, July 2020, Pages 664–671, https://doi.org/10.1093/ageing/afaa015. This is the output from the research you cited on developing accessible statements (reference 39).

Response:

Thank you for this suggestion. We acknowledged the COS mentioned in the comment.

Changes in the manuscript (background):

COS for healthcare interventions in dementia, listed in the database of the Core Outcome Measures in Effectiveness Trials Initiative (COMET) [25], predominately focus on outcomes of persons with dementia, as does the core outcome set for the evaluation of non-pharmacological community-based health and social care interventions for people with dementia living at home [26]. In addition, a set of measures has been recommended to evaluate psychosocial interventions for persons with dementia and their family caregivers [27].

Comment 2:

2. You may wish to consider whether your COS could be considered a bolt on to the one above, as I am aware researchers in more specific fields are currently doing this (and this is being encouraged by COMET).

Response:

Thank you for pointing this out. We have elaborated on our assumptions about how the COS of technology-assisted counselling may be considered in relation to existing COS.

Changes in the manuscript (background):

Non-pharmacological health and social care interventions include psychosocial and psychological interventions, educational and social programmes, case management and care coordination as well as assistive technology [26]. Psychosocial interventions may address persons with dementia, family caregivers, or both and are derived from diverse theories targeting a broad range of outcomes such as well-being, mood, or behaviour [28]. A COS that specifically focuses on technology-assisted counselling interventions for family dementia caregivers may contribute to assess the effectiveness of these interventions and to fill this gap in knowledge. In addition, outcomes associated with the use of ICT in delivering counselling may be identified in the developmental process. By drawing on existing COS, we assume that there will be overlapping domains but also areas, which may be complementary.

Comment 3:

3. The authors may wish to elaborate on their definition of 'minors' on page 6, line 35.

Response:

Thank you for this suggestion. We have replaced the term 'minors' with 'underaged persons' and added an explanation.

Changes in the manuscript (methods and analysis):
Underaged persons (under 18 years old)and persons unable to consent for language reasons will not be included.

Comment 4:
4. The authors may wish to consider giving some indication as to how many participants they intend to recruit for the qualitative sub-study.

Response:
We have included numbers on how many participants we intend to recruit for the qualitative sub-study.

Changes in the manuscript (methods and analysis):
We intend to recruit about 15 caregivers of persons with dementia and about ten persons delivering counselling. The final sample size will be determined by saturation of information during the data collection process [44].

Comment 5:
5. The discussion highlights what the next project will do, namely attain consensus on the long-list and programme theory. I would value a more thorough discussion, including on strengths/limitations, and how the outputs from this fuller programme of work (i.e. when the programme theory and COS are fully developed) will inform further research.

Response:
Thank you for pointing this out. We have included two sections in the manuscript elaboration on possible strengths and limitation and on how the finalised programme theory and COS will inform further research.

Changes in the manuscript (discussion):
Methodological approaches in the ProCOS study follow accepted guidelines [24, 30]. In trying to address shortcomings of previous research, the methodological strands for developing a programme theory and a COS will be integrated. This innovation is a strength of this study, ensuring a theory-led approach to the development, implementation, and evaluation of a technology-assisted counselling intervention for family caregivers of persons with dementia [56].

Logic models have been used to synthesise data in systematic reviews [47–50, 57]; and as a visualisation of programme theory [29, 30] they represent a core element in developing and evaluating complex interventions [29]. It has been debated whether logic models can represent the double complexity resulting from interacting components of complex interventions and from adaptations of interventions to the context [58]. Methodological approaches to develop more dynamic logic models have been proposed [52] and we will reflect upon possible limitations in using logic models to capture double complexity.

(…)

The finalised programme theory will guide the development and implementation of a technology-assisted counselling intervention for family caregivers of persons with dementia. The consented COS will be used to assess the effectiveness of the intervention and may inform further research in the area of technology-assisted counselling in dementia.

VERSION 2 – REVIEW

**REVIEWER**	Brooks, Deborah<br>University of Queensland Centre for Clinical Research
**REVIEW RETURNED**	25-Mar-2024

**GENERAL COMMENTS**	Thank you for addressing the reviewers' comments. I have no further comments for the authors.

**REVIEWER**	Harding, Andrew<br>Lancaster University, Faculty of Health & Medicine
**REVIEW RETURNED**	07-Mar-2024

**GENERAL COMMENTS**	Thank you for making the suggested revisions by the reviewers. I think this is an innovative and interesting protocol, and recommend it for publication.
